# Trithiocyanurate Complexes of Iron, Manganese and Nickel and Their Anticholinesterase Activity

**DOI:** 10.3390/molecules19044338

**Published:** 2014-04-08

**Authors:** Pavel Kopel, Karel Dolezal, Vratislav Langer, Daniel Jun, Vojtech Adam, Kamil Kuca, Rene Kizek

**Affiliations:** 1 Department of Chemistry and Biochemistry, Faculty of Agronomy, Mendel University in Brno, Zemedelska 1, CZ-613 00 Brno, Czech Republic; 2 Central European Institute of Technology, Brno University of Technology, Technicka 3058/10, CZ-616 00 Brno, Czech Republic; 3 Centre of the Region Hana for Biotechnological and Agricultural Research, Faculty of Science, Palacky University & Institute of Experimental Botany, Academy of Sciences of Czech Republic, Slechtitelu 11, CZ-783 71 Olomouc, Czech Republic; 4 Environmental Inorganic Chemistry, Department of Chemical and Biological Engineering, Chalmers University of Technology, SE-412 96 Göteborg, Sweden; 5 Faculty of Military Health Sciences, University of Defence, Trebesska 1575, CZ-50001 Hradec Kralove, Czech Republic; 6 Biomedical Research Center, University Hospital Hradec Kralove, Sokolska 581, CZ-50005 Hradec Kralove, Czech Republic

**Keywords:** trithiocyanuric acid, trimercaptotriazine, crystal structure, complexes, acetylcholinesterase activity, Schiff base

## Abstract

The complexes of Fe(II), Mn(II) and Ni(II) with a combination of a Schiff base, nitrogen-donor ligand or macrocyclic ligand and trithiocyanuric acid (ttcH_3_) were prepared and characterized by elemental analysis and spectroscopies. Crystal and molecular structures of the iron complex of composition [Fe(L_1_)](ttcH_2_)(ClO_4_)·EtOH·H_2_O (**1**), where L_1_ is Schiff base derived from tris(2-aminoethyl)amine and 2-pyridinecarboxaldehyde, were solved. It was found that the Schiff base is coordinated to the central iron atom by six nitrogens forming deformed octahedral arrangement, whereas trithiocyanurate(1-) anion, perchlorate and solvent molecules are not coordinated. The X-ray structure of the Schiff base sodium salt is also presented and compared with the iron complex. The anticholinesterase activity of the complexes was also studied.

## 1. Introduction

The sodium salt of trithiocyanuric acid (ttcH_3_ = trithiocyanuric acid, also named as 2,4,6-trimercapto-1,3,5-triazine (TMT)) readily forms precipitates with heavy metal ions and that is why it is used for removal of heavy metal ions from industrial wastewater. The effectiveness of heavy metal removal was widely studied by Atwood *et al.* [1,2,3] and other groups [4]. Removal of residual palladium and its compounds from reaction mixtures in preparation of drugs, in which palladium is used as a catalyst, is also very important [5,6].

Biological activity of trithiocyanuric compound was also evaluated as it can serve as a ligand of *Toxoplasma gondii* orotate phosphoribosyltransferase [7,8,9]. This enzyme is necessary for replication of the parasitic protozoan *Toxoplasma gondii*, which causes the disease toxoplasmosis. It was proved that trithiocyanuric acid is a better ligand for the enzyme than 5-fluorouracil and emimycin, which are used for clinical treatment of toxoplasmosis. Kar *et al.* prepared a series of trinuclear Ru(II) complexes of a composition [{Ru(L)_2_}_3_(ttc)](ClO_4_)_3_, where L = 2,2'-bipyridine, 1,10-phenanthroline and arylazo-pyridine, which contain trithiocyanurate(3-) bridge bounding Ru(II) centers by chelating S, N donor sets of the anion [10,11]. In addition to the structural, electrochemical and spectral study, interaction of the complexes with the circular and linear forms of p-Bluescript DNA was reported. The Ru(II) complexes reduced the fluorescence intensity of both circular and linear DNA. Zn(II), Fe(II) and Mn(II) complexes with a combination of nitrogen-donor ligands and ttcH_3_ were prepared and their antitumor and antimicrobial activities were assayed [12]. The IC_50_ values of the Fe(II) and Mn(II) compounds turned out to be lower than those of cisplatin and oxaliplatin.

Potentially six donor atoms can be used for coordination to metal centres. It is always difficult to avoid the formation of precipitates of unknown and probably polymeric structure with metal ions in the presence of deprotonated trithiocyanuric acid. Mostly blocking ligands on metal centres must be coordinated. Despite of that, bonding properties of trithiocyanuric acid complexes were proved by single crystal X-ray analysis. In some compounds, only deprotonated trithiocyanuric acid is present as anion not bonded to central atoms [13]. Mononuclear nickel and zinc complexes with nitrogen donor ligands and trithiocyanurate(2-) bonded by S and N have been structurally characterized [14,15,16,17,18]. Bridging, bischelating S, N mode was proved on cobalt complex [{Co(en)_2_}_2_(μ-ttc)](ClO_4_)_3_·2H_2_O (en = ethylenediamine) for the first time [19]. Metals preferring S donor atoms can form trinuclear species with coordination to S atoms only, for example in [{HgMe}_3_(μ-ttc)], [{SnMe_3_}_3_(μ-ttc)] and [{SnPh_3_}_3_(μ-ttc)] [20,21]. The hexanuclear [{AgPPh_3_}_6_(μ-ttc)_2_] complex with two parallel triazine rings held by six Cu-S bridges was characterized [22], as well as the Au(I) cluster [23] and Cu(I) polymer [24]. Trinuclear cyclopentadienyl complexes of rhodium and iridium were also reported [25,26]. Magnetic and structural studies on trinuclear copper complex with 1, 3-bis(2-(4-methylpyridyl)imino)isoindoline as blocking ligand and ttc were reported [27]. Pmdien (*N, N, N',N'', N''*-pentamethyldiethylenetriamine) was proven to be a very good terdentate ligand for complexes with ttc. Trinuclear complexes of compositions [M_3_(pmdien)_3_(μ-ttc)](ClO_4_)_3_, where M = Zn, Cu and Ni were prepared and structurally characterized [28,29,30,31].

**Figure 1:**
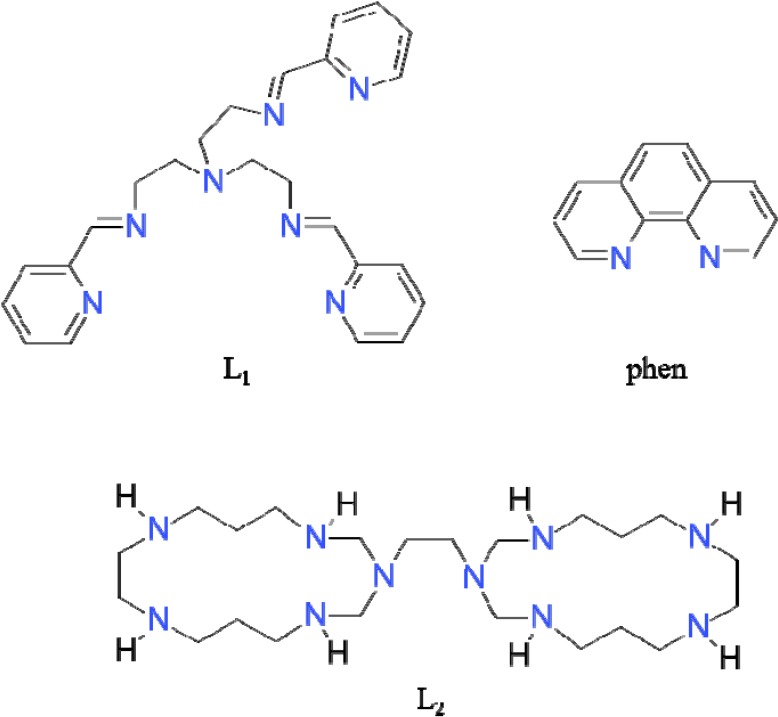
Structural formulas of the ligands used. L_1_ = 2-[(*E*)-2-pyridylmethyleneamino]-*N, N-*bis[2-[(E)-2-pyridylmethyleneamino]ethyl]ethanamine, phen = 1,10-phenanthroline, L_2_ = 3-[2-(1,3,5,9,12-pentazacyclopentadec-3-yl)ethyl]-1,3,5,9,12-pentazacyclopentadecane.

The aim of this work was to prepare Fe(II), Mn(II) and Ni(II) complexes with nitrogen atom donors and trithiocyanurate anion. The complexes are of the following composition: [Fe(L_1_)](ttcH_2_)(ClO_4_)·EtOH·H_2_O (**1**), [Mn_3_(phen)_6_(ttc)](ClO_4_)_3_ (**2**), and Ni_2_(L_2_)(ttcH)(ClO_4_)_2_·6H_2_O·EtOH (**3**), where L_1_ = 2-[(*E*)-2-pyridylmethyleneamino]-*N, N*-bis[2-[(E)-2-pyridylmethyleneamino]ethyl]ethanamine, ttcH_3_ = trithiocyanuric acid, phen = 1,10-phenanthroline and L_2_ = 3-[2-(1,3,5,9,12-pentazacyclopentadec-3-yl)ethyl]-1,3,5,9,12-pentazacyclopentadecane. The structures of the ligands are depicted in Figure 1.

We also attempted to prepare single crystals for X-ray analysis to confirm the stereochemistry of the compounds and characterize them by physico-chemical methods. Due to the presence of a positive charge in the molecule, prepared compounds can interact with the enzyme acetylcholinesterase (AChE; EC 3.1.1.7). Acetylcholine, the natural AChE substrate, contains a positively charged quaternary nitrogen, which is responsible for its interaction with the enzyme active site [32]. Therefore, the other goal of our study was to test the possible anticholinesterase activity of the synthesized complexes.

## 2. Results and Discussion

### 2.1. Synthesis and Spectral Study

**Figure 2:**
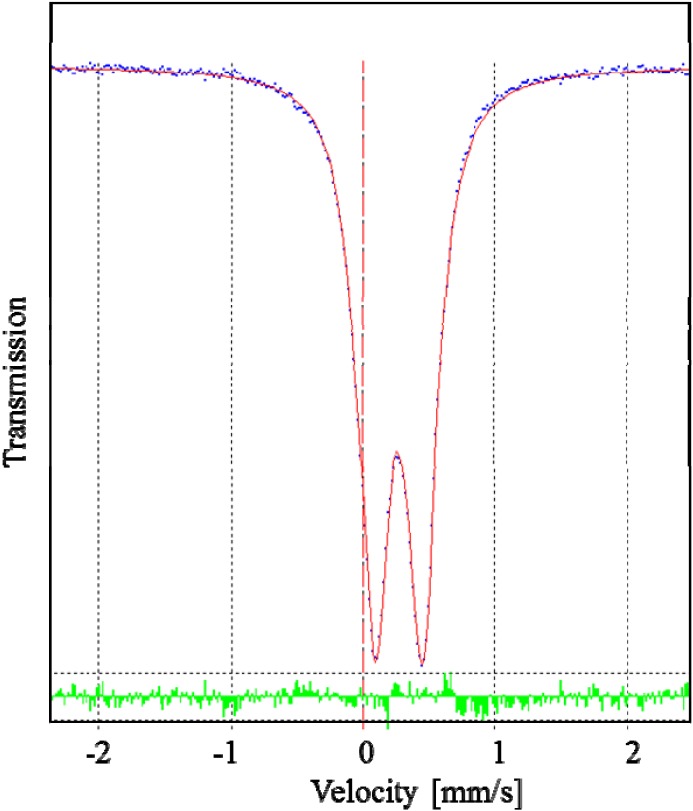
The room temperature Mössbauer spectrum of **1**. The solid line results from least squares fitting of the data to the theoretical equation.

[Fe(L_1_)](ttcH_2_)(ClO_4_)·EtOH·H_2_O (**1**) was prepared by the reaction of iron perchlorate, Schiff base (formed *in situ*), and ttcNa_3_ in an ethanol–water mixture. Although we expected the formation of a binuclear or polynuclear complex with a trithiocyanurate bridge, only a mononuclear Fe(II) complex was formed. Its composition was proposed on the base of elemental analysis and unambiguously confirmed by single crystal X-ray analysis. The deformed octahedral coordination of the central Fe(II) was also confirmed by Mössbauer spectroscopy (see Figure 2). The room temperature Mössbauer spectrum of **1** is composed of two doublets with the isomer shift values (0.28 and 0.14 mm s^−1^) typical of octahedral low-spin iron(II) complexes [33,34]. The doublet *I* with relative spectrum area *A* = 91.4% has a higher value of the quadrupole splitting parameter (q.s. = 0.28 mm s^−1^) than the doublet *II* (*A* = 8.6%, q.s. = 0.20 mm s^−1^). The two different values of quadrupole splitting show that there are two octahedrally coordinated iron centers with lower and higher distortion from the ideal octahedral arrangement, found in the polycrystalline material, but one arrangement is dominant. Similar spectra with two doublets were also found as a result of the octahedral arrangement distortion of the central atoms in Fe(II) complexes [12,13]. Our attempts to prepare Schiff base L_1_ in solid form were unsuccessful, but finally its sodium complex Na(L_1_)ClO_4_ (**4**) was obtained from the reaction mixture as light-yellow crystals, suitable for X-ray study. Complex **4** was also obtained by the reaction of L_1_ with sodium perchlorate. Its structure is discussed hereinafter.

The complex [Mn_3_(phen)_6_(ttc)](ClO_4_)_3_ (**2**) was prepared according to Cermakova [35]. The complex was characterized by FTIR and Raman spectroscopies, MALDI-TOF mass spectrometry, magnetic and conductivity measurements. On the basis of different techniques, the trinuclear structure of complex was proposed, where three central manganese atoms are connected by trithiocyanurate(3-) bridge.

Complex Ni_2_(L_2_)(ttcH)(ClO_4_)_2_·6H_2_O·EtOH (**3**) was prepared from Ni(bapen)(ClO_4_)_2_ (bapen = *N, N'-*bis(3-aminopropyl)ethylenediamine) and *in situ* formation of macrocyclic ligand L_2_ by the condensation reaction of the terminal amino groups of bapen and ethylenediamine with formaldehyde. A similar preparation of macrocyclic complexes was for example published by Comba *et al.* [36]. Addition of ttcNa_3_ led to a formation of violet crystalline product. As our attempts to prepare crystals for X-ray analysis were unsuccessful we used mass spectroscopy to confirm the composition of **3**.

The ESI^−^ mass spectra displays intense peaks at *m/z* = 947 and *m/z* = 848, corresponding to the binuclear molecular ion with ClO_4_^−^ adducts of composition [Ni_2_(L_2_)(ttcH)(ClO_4_)_2_H^−^]^−^ and [Ni_2_(L_2_)(ttcH)(ClO_4_)H^−^]^−^, respectively. The formation of perchlorate ion adducts is well known for such kinds of complex ions [31,37]. The peaks observed at lower *m/z* = 514, 455, 233 and 99, correspond to different fragments of the complex and its organic parts.

The value of effective magnetic moment calculated per nickel(II) (μ_eff_ = 3.31 BM) for **3** is higher than that expected for the spin only value of octahedral or pentacoordinated nickel central atoms (μ_so_ = 2.83 BM). The higher value of the magnetic moment can be explained by a spin-orbital contribution to the spin only value. We can assume that the central nickel atoms are coordinated by four N atoms of macrocyclic ligand and by N or N, S set of donor atoms of ttcH^−^ anion.

### 2.2. X-ray Structures of [Fe(L_1_)](ttcH_2_)(ClO_4_)·EtOH·H_2_O (**1**) and Na(L_1_)ClO_4_ (**4**)

**Figure 3:**
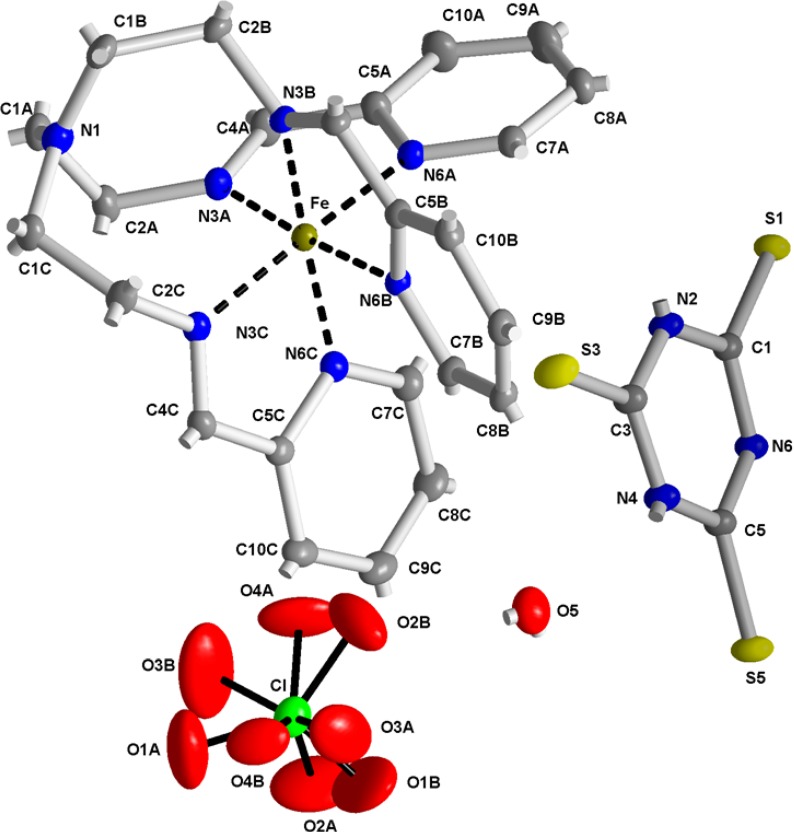
Numbering scheme of **1** with atomic displacement ellipsoids drawn at 30% probability level. Hydrogen atoms are omitted for clarity. Note the disordered perchlorate anion.

**Figure 4:**
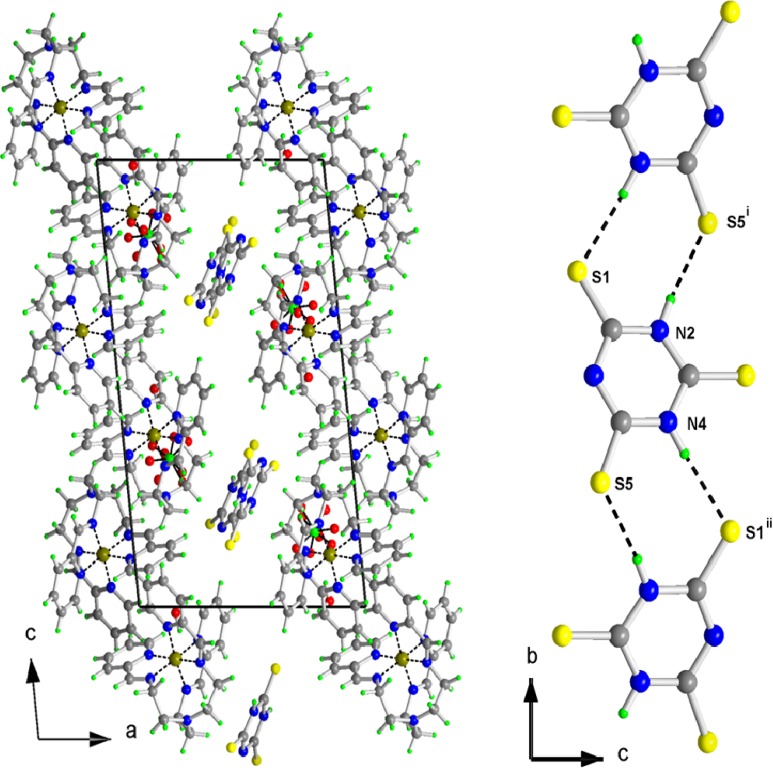
Projection of the contents of the unit cell along b-axis (on **left**). Note the chains of ttc along the b-axis (on **right**).

The molecular structure of [Fe(L_1_)](ttcH_2_)(ClO_4_)·EtOH·H_2_O (**1**) is depicted in Figure 3, while selected bond lengths and angles are listed in Table 1. The crystal structure is stabilized by hydrogen bonds (see Table 2, Figure 4). The molecular structure of **1** consists of an electroneutral iron(II) complex, ttcH^2−^ and ClO_4_^−^ anions [disordered at two orientations with occupancies 0.536(8)/0.464(8)] and crystal water and EtOH molecules (mathematically squeezed of due to a disorder which could not be properly modeled). The central iron atom is coordinated by six N atoms of Schiff base L_1_ in a deformed octahedral arrangement. The bond lengths of the azomethine nitrogens N3A, N3B and N3C to central atom are in the range of 1.9527(16)-1.9635(16) Å, whereas the bond lengths of the pyridine nitrogens N6A, N6B and N6C are significantly longer (1.9720(16)-1.9997(15) Å). The N1 atom is out the coordination sphere of Fe atom with distance 3.4576(18) Å.

**Figure 5:**
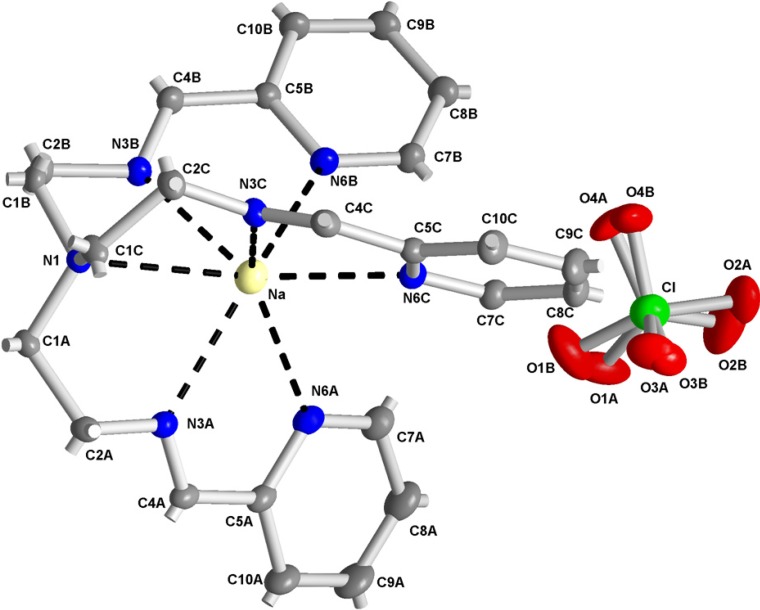
Numbering scheme of **4** with atomic displacement ellipsoids drawn at 30% probability level. Hydrogen atoms are omitted for clarity. Note the disordered perchlorate anion.

The molecular structure of Na(L_1_)ClO_4_ (**4**) is depicted in Figure 5, while selected bond lengths and angles are listed in Table 3. Again, the perchlorate was disordered with occupancies 0.48(2)/0.52(2). The bond lengths to azomethine nitrogens are again shorter [2.580(14)-2.5406(13) Å] than those to pyridine nitrogens [2.6224(13)-2.7353(14) Å]. Moreover, atom N1 is in the coordination sphere of sodium, so Na is coordinated by seven N atoms.

**Table 1:** Selected bond lengths [Å] and angles [°] for **1**.

Fe-N3B	1.9527(16)	N3B-Fe-N3C	96.12(7)	N3C-Fe-N6A	174.37(7)
Fe-N3A	1.9569(16)	N3A-Fe-N3C	96.15(7)	N6C-Fe-N6A	94.01(7)
Fe-N3C	1.9635(16)	N3B-Fe-N6C	171.87(7)	N3B-Fe-N6B	80.89(6)
Fe-N6C	1.9720(16)	N3A-Fe-N6C	91.45(7)	N3A-Fe-N6B	175.87(7)
Fe-N6A	1.9764(16)	N3C-Fe-N6C	81.10(7)	N3C-Fe-N6B	87.31(6)
Fe-N6B	1.9997(15)	N3B-Fe-N6A	89.10(7)	N6C-Fe-N6B	91.32(6)
N3B-Fe-N3A	96.45(7)	N3A-Fe-N6A	81.11(7)	N6A-Fe-N6B	95.64(6)

**Table 2:** Hydrogen bonds for 1 [Å, °].

D-H...A	d(D-H)	d(H...A)	d(D...A)	<(DHA)
O5-H52...O2B	0.931(18)	1.96(2)	2.825(7)	153
O5-H52...O4A	0.931(18)	2.35(2)	3.243(10)	160
O5-H52...O1B	0.931(18)	2.54(3)	3.334(9)	144
O5-H52...Cl	0.931(18)	2.84(2)	3.765(2)	171
N2-H2...S5^i^	0.88	2.52	3.3879(17)	169
N4-H4...S1^ii^	0.88	2.40	3.2418(17)	160

Symmetry transformations used to generate equivalent atoms: (i): −x+1, y +1/2,−z−1/2; (ii): −x+1,y−1/2,−z−1/2.

**Table 3:** Selected bond lengths [Å] and angles [°] for **4**.

Na-N3A	2.5280(14)	N3A-Na-N6C	115.01(5)	N6C-Na-N6A	88.66(4)
Na-N3C	2.5338(13)	N3C-Na-N6C	64.88(4)	N6B-Na-N6A	90.08(4)
Na-N3B	2.5406(13)	N3B-Na-N6C	141.07(5)	N3A-Na-N1	65.01(4)
Na-N6C	2.6224(13)	N3A-Na-N6B	145.06(5)	N3C-Na-N1	64.31(4)
Na-N6B	2.6300(14)	N3C-Na-N6B	110.99(5)	N3B-Na-N1	65.04(4)
Na-N6A	2.7353(14)	N3B-Na-N6B	65.02(4)	N6C-Na-N1	127.09(4)
Na-N1	2.8431(12)	N6C-Na-N6B	84.78(4)	N6B-Na-N1	126.93(4)
N3A-Na-N3C	103.63(4)	N3A-Na-N6A	63.49(4)	N6A-Na-N1	126.25(4)
N3A-Na-N3B	103.60(4)	N3C-Na-N6A	143.27(5)	N6C-Na-N6A	88.66(4)
N3C-Na-N3B	102.28(4)	N3B-Na-N6A	114.01(4)		

**Figure 6:**
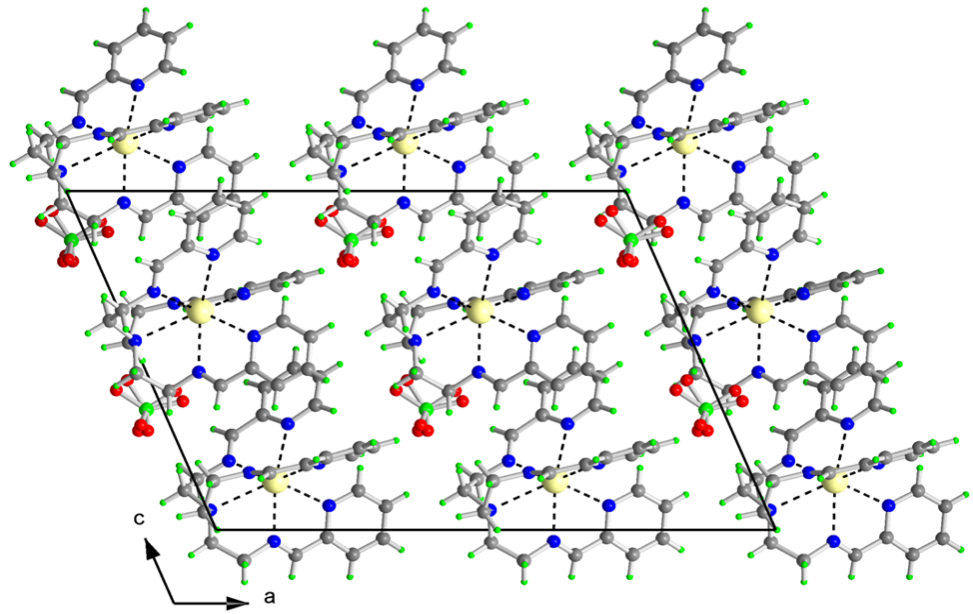
Projection of the contents of the unit cell along b-axis for **4**.

The absolute configuration with Flack parameter −0.03(4) was determined. The crystal structure is stabilized by weak hydrogen bonds (see Table 4, Figure 6). Dihedral angles between the pyridine rings A, B and C are 89.70(10), 75.41(9) and 82.48(10) degrees for **1** and 53.78(8), 67.49(8) and 68.95(9) degrees for **4**, respectively.

**Table 4:** Hydrogen bonds for **4** [Å, °].

D-H...A	d(D-H)	d(H...A)	d(D...A)	<(DHA)
C2B-H2B1...O3B^i^	0.99	2.56	3.483(11)	154
C7A-H7A...O1B	0.95	2.59	3.264(10)	128

Symmetry transformations used to generate equivalent atoms: (i): x−1/2, y−1/2,z.

### 2.3. Anticholinesterase Activity

**Figure 7:**
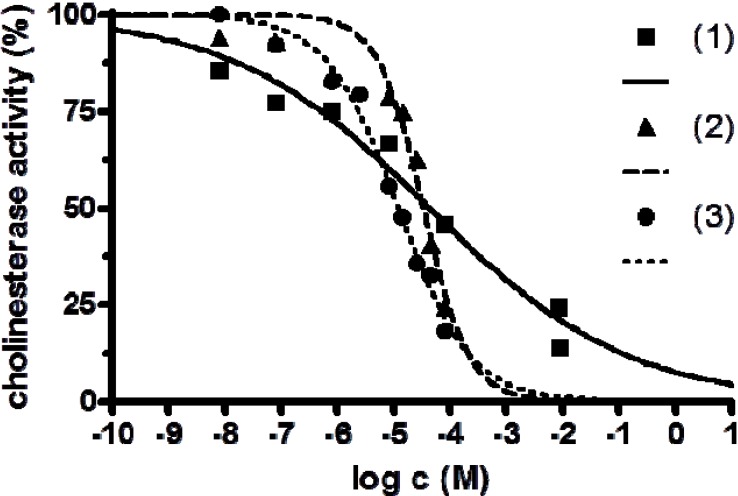
Anticholinesterase activity of complexes [Fe(L_1_)](ttcH_2_)(ClO_4_)·EtOH·H_2_O (**1**), [Mn_3_(phen)_6_(ttc)](ClO_4_)_3_ (**2**) and Ni_2_(L_2_)(ttcH)(ClO_4_)_2_·6H_2_O·EtOH (**3**).

The anticholinesterase activity of the complexes **1**–**3** and Fe(II), Mn(II) and Ni(II) salts were studied. The results of the study are presented in Table 5 and in Figure 7. As it is clearly seen, the newly prepared complexes of Fe(II) and Mn(II) were more than one hundred times and Ni(II) complex one thousand times stronger inhibitors if compared with corresponding standards (FeSO_4_, MnSO_4_, NiSO_4_).

All the complexes show low solubility in water and are well soluble in DMF and DMSO. From the composition of [Fe(L_1_)](ttcH_2_)(ClO_4_)·EtOH·H_2_O (1) proved by X-ray it is obvious that in solution, complex cation and ttcH_2_ and ClO_4_ anions are formed. The complex cation is very stable due to the chelating Shiff base on the iron(II) center as was demonstrated, for example, in a study of oxygen bridged [(salen)FeOFe(salen)] (H_2_salen = *N, N'*- bis(salicylidene)ethylene diamine) complex [38]. Also in complexes [Fe(bpy)_3_](ttcH)·2bpy·7H_2_O and [Fe(phen)_3_](ttcH_2_)(ClO_4_)·2CH_3_OH·2H_2_O, where bpy = 2,2'-bipyridine, phen = 1,10-phenanthroline, the strong N-N ligands prevent the coordination of ttc anion to the metal center [13]. In the case of 1, we can assume that biological activity is caused by a combined effect of the individual components presented within the corresponding mixture in the medium used.

**Table 5:** Anticholinesterase activity of prepared complexes and standards *in vitro*.

Compound	IC_50_ [M]	95% CI [M]	HillSlope
[Fe(L_1_)](ttcH_2_)(ClO_4_)·EtOH·H_2_O ( **1**)	4.35 × 10^−5^	1.34 × 10^−5^–1.41 × 10^−4^	0.0285
[Mn_3_(phen)_6_(ttc)](ClO_4_)_3_ ( **2**)	3.34 × 10^−5^	2.28 × 10^−5^–4.88 × 10^−5^	0.2280
Ni_2_(L_2_)(ttcH)(ClO_4_)_2_·6H_2_O·EtOH ( **3**)	1.20 × 10^−5^	9.46 × 10^−6^–1.51 × 10^−5^	0.0552
FeSO_4_	>10^−3^	-	-
MnSO_4_	>10^−3^	-	-
NiSO_4_	1.29 × 10^−2^		−0.3920

We can assume the stability of [Mn_3_(phen)_6_(ttc)](ClO_4_)_3_ (**2**) from the MALDI-TOF mass spectra, where the molecular peak was found [35]. Fragments of the complex were present in the spectra but we can expect that once the ttc bridge is formed it is bonded to the metal centre. We proved this by our attempt to prepare single crystals of **2**. We dissolved the complex in DMSO and added diethyl ether to induce crystallization. After two weeks, we have only obtained from the solution single crystals of the dinuclear complex [Mn_2_(phen)_4_(ttc)](ClO_4_) (its structure will be published elsewhere), so it can be suggested that the complex undergoes dissociation but it can be considered as stable enough for biological activity testing. The complex Ni_2_(L_2_)(ttcH)(ClO_4_)_2_·6H_2_O·EtOH (**3**) was studied by ESI^−^ mass spectroscopy and a molecular peak was found. Macrocyclic ligands form very stable complexes as it can be demonstrated on multinuclear zinc cyclen (1,4,7,10-tetraazacyclododecane) complexes with ttc bridges, which are stable in water at neutral pH [39]. In this case of **3**, the biological activity is caused either by a combined effect of macrocyclic complex and ttc anion or by complex with coordinated ttc ligand.

Due to the data obtained, further investigation of the anticholinesterase activity of the prepared complexes should be done. Because of the potency of tested compounds to inhibit cholinesterases, it could be considered to design structurally related complexes as potential drugs for Alzheimer´s disease or as prophylactics in case of nerve agent or pesticide poisoning.

## 3. Experimental Section

### 3.1. Materials and Methods

Safety note: *Caution!* Perchlorate salts of metal complexes with organic ligands are potentially explosive and should be handled with great care. The chemicals and solvents were supplied by Aldrich (St. Louis, MO, USA) and used without further purification. The C, H, N, and S analyses were carried out on an EA 1108 instrument (Fisons Instruments, Rodano, Italy). The magnetochemical data were obtained by Faraday method at 293 K using a M-25D electrobalance (Sartorius, Elk Grove, IL, USA). Hg[Co(SCN)_4_] was used as a calibrant. The correction for diamagnetism was calculated using Pascal’s constants. The transmission Mössbauer spectrum was recorded using a Mössbauer spectrometer in constant acceleration mode with a ^57^Co(Rh) source. Isomer shift parameters are related to metallic iron (the calibration temperature of 300 K).

The ESI^-^ mass spectra were recorded on a ZMD 2000 mass spectrometer (Waters, Milford, MA, USA). The mass-monitoring interval was *m/z* 10–1500. The spectra were collected using 3.0 s cyclical scans and applying the sample cone voltages 20, 30 or 40 V, at the source block temperature 80 °C, desolvation temperature 150 °C and desolvation gas flow rate 200 l/h. The mass spectrometer was directly coupled to a MassLynx data system. All *m/z* interpretations were based on ^35^Cl and ^58^Ni, respectively.

The crystallographic data for the structures **1** and **4** has been deposited with the Cambridge Crystallographic Data Centre as supplementary publication no. 960842 and 960843. Copies of the data can be obtained, free of charge, on application to CCDC, 12 Union Road, Cambridge, CB2 1EZ, UK (fax: +44-(0)1223-336033 or e-mail: deposit@ccdc.cam.ac.uk).

### 3.2. Preparation of the Complexes

#### 3.2.1. [Fe(L_1_)](ttcH_2_)(ClO_4_)·EtOH·H_2_O (**1**)

Schiff base L_1_ was prepared *in situ* by condensation of tris(2-aminoethyl)amine (150 µL, 1 mmol) and 2-pyridinecarboxaldehyde (285 µL, 3 mmol) in EtOH (30 mL). The mixture was heated to boiling and after cooling, added to stirred EtOH solution (30 mL) of Fe(ClO_4_)_2_·6H_2_O (0.36 g, 1 mmol). The violet mixture was heated at 60 °C for 30 min. A little amount of precipitate disappeared after addition of water (20 mL). After cooling, ttcNa_3_·9H_2_O (0.4 g, 1 mmol) in water (5 mL) was added dropwise to the solution. A small amount of precipitate was filtered off. A dark violet solution was left for the crystallization. After a week, violet crystals suitable for X-ray analysis were collected. Yield: 62%. Anal. Calcd.: C, 43.1; H, 4.6; N, 17.3; S, 11.9. Found: C, 42.8; H, 4.5; N, 17.1; S, 11.3%. Mössbauer spectrum (300 K): doublet *I* with isomer shift (i.s.) = 0.28 ± 0.01 mm s^−1^, quadrupole splitting (q.s.) = 0.18 ± 0.00 mm s^−1^, half-width of the spectral line (Γ) = 0.26 ± 0.01 mm s^−1^ and relative spectrum area (A) = 91.4%; doublet *II* with i.s. = 0.14 ± 0.01 mm s^−1^, q.s. = 0.20 ± 0.01 mm s^−1^, Γ = 0.23 ± 0.01 mm s^−1^ , A = 8.6%.

#### 3.2.2. [Mn_3_(phen)_6_(ttc)](ClO_4_)_3_ (**2**)

The complex was prepared according to [35]. 1, 10-Phenanthroline (phen) (0.4 g, 2 mmol) in EtOH (15 mL) was added to an EtOH solution (15 mL) of manganese(II) perchlorate hexahydrate (0.36 g, 1 mmol). The yellow precipitate was dissolved by addition of water (40 mL). Then, ttcNa_3_·9H_2_O (0.14 g, 0.35 mmol) in water (1 mL) was added in drops to the solution. The yellow precipitate was filtered off, washed several times with water and EtOH and dried at 60 °C. Yield: 78%. Anal. Calcd.: C, 52.4; H, 2.8; N, 12.2; S, 5.6. Found: C, 51.8; H, 2.9; N, 12.1; S, 5.2%.

#### 3.2.3. Ni_2_(L_2_)(ttcH)(ClO_4_)_2_·6H_2_O·EtOH (**3**)

*N, N'*-bis(3-aminopropyl)ethylenediamine (bapen) (0.55 mL, 3 mmol) was added to an EtOH solution (100 mL) of Ni(ClO_4_)_2_·6H_2_O nickel(II) perchlorate hexahydrate (1.11 g, 3 mmol) and stirred for 1 h. Triethylamine (0.84 mL, 6 mmol), ethylenediamine (0.1 mL, 1.5 mmol) and paraformaldehyde (0.18 g, 6 mmol) were added and the mixture was stirred and refluxed for 24 h. The mixture was filtered while hot and the solution was left to cool to room temperature. A solution of ttcNa_3_·9H_2_O (0.2 g, 0.5 mmol) in water (5 mL) was added in form of drops. The colour of the solution turned to dark violet and microcrystals of the product were collected after 3 h on a frit, washed several times with EtOH and dried in air. Yield: 47%. Anal. Calcd.: C, 29.4; H, 6.5; N, 16.5; S, 8.7. Found: C, 28.8; H, 6.1; N, 16.2; S, 8.1%. MS (ESI^−^): *m/z* = 947 [Ni_2_(L_2_)(ttcH)(ClO_4_)_2_H^−^]^−^, 848 [Ni_2_(L_2_)(ttcH)(ClO_4_)H^−^]^−^, 514 [Ni(L_2_)H^−^]^−^, 455 [(L_2_)H^−^]^−^, 233 [Ni(ttcH)H^−^]^−^, 99 ClO_4_^−^. μ_eff_ = 3.31 BM.

#### 3.2.4. Na(L_1_)ClO_4_ (**4**)

Schiff base L_1_ was prepared by condensation of tris(2-aminoethyl)amine (150 µL, 1 mmol) and 2-pyridinecarboxaldehyde (285 µL, 3 mmol) in EtOH (30 mL). The mixture was heated to boiling and after cooling EtOH solution (2 mL) of NaClO_4_·H_2_O (0.14 g, 1 mmol) was added. Obtained precipitate was collected on frit, washed with EtOH and dried at 40 °C. Yield: 53%. Anal. Calcd.: C, 53.8; H, 5.1; N, 18.3. Found: C, 53.2; H, 4.9; N, 18.1%.

### 3.3. X-ray Crystallography

X-ray data of **1** and Na(L_1_)ClO_4_ (**4**) were collected on a SMART CCD diffractometer (Siemens, Madison, WI, USA) with Mo-Kα radiation (λ = 0.71073 Å, graphite monochromator). The crystal was cooled to 173(2) K by a flow of nitrogen gas using the LT-2A device. A full sphere of reciprocal space was scanned by 0.3 steps in ω with a crystal-to-detector distance of 3.97 cm. Preliminary orientation matrices were obtained from the first frames using SMART [40]. The collected frames were integrated using the preliminary orientation matrix which was updated every 100 frames. Final cell parameters were obtained by refinement of the positions of reflections with I > 10σ (I) after integration of all the frames using SAINT software [40]. The data were empirically corrected for absorption and other effects using the SADABS program [41]. The structures were solved by direct methods and refined by full-matrix least squares on all |F^2^| data using SHELXTL software [42].

X-ray data of **1**: The largest peak and hole on the final difference map were 0.846 and −0.467 e.Å^−3^. Important crystallographic parameters are as follows: C_29_H_37_ClN_10_O_6_S_3_Fe, wavelength 0.71073 Å, monoclinic, space group P2_1_/c, a = 12.5826(7), b = 12.4592(7), c = 22.2479(13) Å, β = 96.098(1)°, volume 3468.0(3) Å^3^, Z = 4, density (calc.) 1.550 Mg/m^3^, absorption coefficient 0.751 mm^−1^, F(000) = 1680, crystal size 0.42 × 0.42 × 0.10 mm, index ranges −17 ≤ *h* ≤ 17, −17 ≤ *k* ≤ 17, −30 ≤ *l* ≤ 30, reflections collected/independent 51041/9351 (*R*_int_ = 0.0369), refinement method full-matrix least-squares on *F*^2^, data/restraints/parameters 9351/71/499, goodness-of fit on *F*^2^ = 1.016, final *R*_1_ (I > 2σ(I) data) = 0.0384, *wR*_2_ = 0.1064, final *R*_1_ (all data) = 0.0501, *wR*_2_ = 0.1120.

X-ray data of **4**: The largest peak and hole on the final difference map were 0.367 and −0.205 e.Å^−3^. Important crystallographic parameters are as follows: C_24_H_27_N_7_NaClO_4_, wavelength 0.71073 Å, monoclinic, space group Cc, a = 21.8301(8), b = 8.5329(3), c = 15.1592(5) Å, β = 112.719(1)°, volume 2604.67(16) Å^3^, Z = 4, density (calc.) 1.367 Mg/m^3^, absorption coefficient 0.208 mm^−1^, F(000) = 1120, crystal size 0.32 x 0.22 x 0.12 mm, index ranges −33 ≤ *h* ≤ 33, −13 ≤ *k* ≤ 13, −22 ≤ *l* ≤ 22, reflections collected/independent 22608/9247 (*R*_int_ = 0.0238), refinement method full-matrix least-squares on *F*^2^, data/restraints/parameters 9247/70/399, goodness-of fit on *F*^2^ = 1.027, final *R*_1_ (I > 2σ(I) data) = 0.0396, *wR*_2_ = 0.0967, final *R*_1_ (all data) = 0.0513, *wR*_2_ = 0.1025.

### 3.4. Biological Activity Testing

*In vitro* inhibition test was conducted as described earlier [43]. Solutions of the prepared Fe(L_1_)](ttcH_2_)(ClO_4_)·EtOH·H_2_O (**1**), [Mn_3_(phen)_6_(ttc)](ClO_4_)_3_ (**2**), and Ni_2_(L_2_)(ttcH)(ClO_4_)_2_·6H_2_O·EtOH (**3**) complexes of appropriate concentrations (concentration range from 10^−7^ to 10^−1^ M; 500 µL) were added to the suspension of rat brain homogenate (10 *w/v* in distilled water; 500 µL), solution of sodium chloride (3 M; 2.5 mL) and water (20 mL). Subsequently, a solution of acetylcholine iodide (0.02 M; 2.0 mL) was added (=starting of the enzyme reaction). The enzyme activity was immediately determined using of automatic titrator RTS 822 (Radiometer, Bronshoj, Denmark). The IC_50_ values were calculated from a plot of percent inhibition of cholinesterases versus its concentration. To show that not only Fe(II), Mn(II) or Ni(II) cation in complex causes the anticholinesterase activity, FeSO_4,_ MnSO_4_ and NiSO_4_ were used as standards for comparison. Nonlinear regression was performed using software for statistical analysis GraphPad Prism version 4 for Windows (GraphPad Software, San Diego, CA, USA; www.graphpad.com).

## 4. Conclusions

Metal based complexes play important roles in numerous applications, including drugs. Their effects on enzyme pathways can be reversible and/or irreversible, which is of great interest for physicians, because such compounds can help alter disease-connected pathways. In this study, we prepared and characterized complexes of Fe(II), Mn(II) and Ni(II) with a combination of Schiff base, nitrogen-donor ligand or macrocyclic ligand and trithiocyanuric acid (ttcH_3_). Besides their structural characterization, their effect on anticholinesterase activity was also examined.
